# Sleep and intrusive memories immediately after a traumatic event in emergency department patients

**DOI:** 10.1093/sleep/zsaa033

**Published:** 2020-03-05

**Authors:** Kate Porcheret, Lalitha Iyadurai, Michael B Bonsall, Guy M Goodwin, Sally A Beer, Melanie Darwent, Emily A Holmes

**Affiliations:** 1 Nuffield Department of Clinical Neuroscience, University of Oxford, Oxford, UK; 2 Turner Institute for Brain and Mental Health, Monash University, Melbourne, Australia; 3 Department of Psychiatry, University of Oxford and Oxford Health NHS Foundation Trust, Oxford, UK; 4 Department of Zoology and St. Peter’s College, University of Oxford, Oxford, UK; 5 Emergency Department, Oxford University Hospitals NHS Foundation Trust, Oxford, UK; 6 Department of Psychology, Uppsala University, Uppsala, Sweden; 7 Department of Clinical Neurosciences, Karolinska Institutet, Uppsala, Sweden

**Keywords:** sleep, mental imagery, intrusive memories, trauma, posttraumatic stress disorder, single symptom

## Abstract

**Study objectives:**

Intrusive memories of psychological trauma are a core clinical feature of posttraumatic stress disorder (PTSD), and in the early period post-trauma may be a potential target for early intervention. Disrupted sleep in the weeks post-trauma is associated with later PTSD. The impact of sleep and intrusive memories immediately post-trauma, and their relation to later PTSD, is unknown. This study assessed the relationship between sleep duration on the first night following a real-life traumatic event and intrusive memories in the subsequent week, and how these might relate to PTSD symptoms at 2 months.

**Methods:**

Patients (*n* = 87) recruited in the emergency department completed a sleep and intrusive memory diary from the day of their trauma and for the subsequent week, with optional actigraphy. PTSD, anxiety, and depression symptoms were assessed at 1 week and 2 months.

**Results:**

A U-shaped relationship was observed between sleep duration on the first night and intrusive memories over the subsequent week: sleeping “too little” or “too much” was associated with more intrusive memories. Individuals who met Clinician-Administered PTSD Scale (CAPS) criteria for PTSD at 2 months had three times more intrusive memories in the first week immediately post-trauma than those who did not (*M* = 28.20 vs 9.96). Post hoc analysis showed that the absence of intrusive memories in the first week post-trauma was only observed in those who did not meet CAPS criteria for PTSD at 2 months.

**Conclusions:**

Monitoring intrusive memories and sleep in the first week post-trauma, using a simple diary, may help identify individuals more vulnerable to later psychopathology.

**Statement of Significance**

We know little about symptoms *immediately* after psychological trauma. This paper examines a core clinical symptom—intrusive memories of trauma—and sleep in patients recruited in the emergency department. Patients who slept “too much” or “too little” on their first night post-trauma reported more intrusive memories in the subsequent week. Furthermore, patients meeting criteria for posttraumatic stress disorder 2 months post-trauma had approximately three times more intrusive memories in this first week post-trauma. Monitoring sleep and intrusive memories in the first week post-trauma using a simple diary may help identify individuals who could benefit from clinical support.

## Introduction

We know little about symptoms *immediately* after a psychologically traumatic event and how these may relate to longer-term psychopathology. One symptom that may be a potential target for preventative interventions immediately after trauma is intrusive memories of the traumatic event. Intrusive memories are vivid, distressing mental images of the event that spring back to mind involuntarily—for example, vividly seeing in the mind’s eye the white flash of an airbag at the moment of a car crash (mental imagery). Intrusive memories of a traumatic event comprise a core clinical feature of both acute stress disorder (ASD, from 3 days to 1 month after a trauma) and posttraumatic stress disorder (PTSD, from 1 month after a trauma) [1]. Intrusive memories, as a single symptom (as opposed to a full diagnosis), can be significantly distressing and disrupt everyday functioning as early as the first week after a traumatic event, whether or not someone has a diagnosis of ASD or PTSD [2]. Furthermore, in the early period (on average 7 days) after trauma, intrusive memories have been centrally linked to other PTSD symptoms [3]. Indeed, cognitive models of PTSD place intrusive memories at the center of the disorder [4], potentially driving other symptoms, such as avoidance, negative alterations in cognitions and mood, and hyperarousal. Consequently, it has been suggested that targeting intrusive memories with early interventions may prevent PTSD from developing [5].

Sleep on the first night after a traumatic event may be linked to the development of intrusive memories. Memory consolidation refers to the time-dependent process by which new memories become stable, lasting memories [6]. Sleep enhances the consolidation of memory, especially emotional memory, in most [7–12], but not all [13], studies. Emotional memory has been postulated to comprise a declarative core of the fact/events of the memory and an emotional component of the feelings and emotions related to that memory [14]. Sleep, especially Rapid Eye Movement (REM) sleep, has been associated with increased consolidation of the declarative core; however, findings are mixed with regard to the emotional component, with REM sleep being associated with both its downregulation [15–19] and enhancement [20–23]. The consolidation of memory is thought to occur over a time window of several hours after the event occurs [6]. This, along with the majority of studies assessing sleep within hours of memory encoding (e.g. [7–12]), suggest that the first sleep episode following an event may be the most important for its consolidation. Since intrusive memories are emotional memories of a traumatic event, it has been hypothesized that preventing sleep after a trauma could weaken trauma memories [24, 25].

We have shown in a previous study that fewer intrusive memories to an analogue traumatic event (trauma film) were reported over the subsequent week by individuals sleep deprived on the first night following the trauma film, compared to those who slept as usual [26]. However, subsequent studies have provided mixed findings: total sleep deprivation at home on the first night following a trauma film resulted in fewer intrusive memories on the first day (but not over the first week) after the film, compared to sleep as usual [27]; women who remained awake (either by staying awake during the day or being sleep deprived overnight) reported more intrusive memories compared to those allowed to sleep as usual overnight [28]; and finally, individuals who briefly napped immediately following a trauma film reported fewer intrusive memories compared to those who stayed awake [29]. Although different methodologies make direct comparisons between these studies tentative, they have all shown a difference in the number of intrusive memories reported between individuals allowed to sleep as usual (or nap) and those who stayed awake for at least 12 h following an analogue trauma, albeit in different directions. Animal models also highlight the importance of sleep on the first night following a traumatic event. In rats, freezing to trauma cues and anxiety-like behavior (an index of time spent in the open arm on the elevated plus maze) were attenuated by total sleep deprivation during the first sleep phase (i.e. during the day for nocturnal animals) compared to sleep [30, 31]. Freezing behavior in a fear-associated memory test during extinction recall was also correlated to less REM sleep [32]. In humans, following a real-life trauma, sleep disturbance within the first weeks or months is associated with the development of later PTSD [33–35], and recently sleep disturbance in the first week post-trauma has been associated with having more intrusive memories in that first week, albeit in a post hoc analysis [36]. What remains unknown is the relationship between sleep *on the first night* following a real-life traumatic event and intrusive memories in the first week post-trauma. Empirical prospective assessment of sleep and intrusive memories immediately following a real-life traumatic event is lacking.

As postulated, if sleep on the first night post-trauma is important for the consolidation of the trauma memory, then it may be relevant in the development of later PTSD, as well as early intrusive memories. To date, no studies to our knowledge have examined the relationship between sleep on the first night after a trauma and later PTSD. Furthermore, little is known about the relationship between the number of intrusive memories experienced in the first week from immediately after a trauma and later PTSD, despite the fact that early intrusive memories have been raised as a possible prevention target. This is important given that the assumption underlying recent early intervention studies [37, 38] is that reducing the number of intrusive memories in the first week post-trauma will be beneficial in reducing the likelihood of later PTSD symptoms.

The primary aim of this study was to examine the relationship between sleep duration on the first night following a real-life traumatic event and the number of intrusive memories over the subsequent week, in patients attending a hospital emergency department (ED) on the day of their trauma. Additional exploratory aims were to investigate the relationship between (1) sleep duration on the first night after the trauma and PTSD symptoms 2 months post-trauma and (2) the number of intrusive memories in the first week post-trauma and PTSD symptoms 2 months post-trauma.

## Methods

### Participants

Patients presenting to the ED of the John Radcliffe Hospital, Oxford, UK were screened for eligibility by research nurses (weekdays, 08:00 am to 08:00 pm, November 2016 to December 2017; *n* = 976). Inclusion criteria were age at least 18 years, experienced or witnessed a traumatic event (i.e. exposure to actual or threatened death, serious injury, or sexual violence), presented to the ED on the same day as the traumatic event, reported memory of the event, fluent in written and spoken English, and alert and orientated (Glasgow Coma Scale score = 15). Exclusion criteria were loss of consciousness, history of severe mental illness, current intoxication, substance abuse, or neurological condition, or currently suicidal.

Eligibility was assessed using medical records and face-to-face interviews. Potentially eligible patients (*n* = 162) were approached about the study, and 62 patients declined to take part. After receiving a description of the study, 100 participants provided written informed consent. One participant was excluded from the study post hoc, due to the disclosure of a neurological condition, giving a final eligible sample of 99 participants.

Participant characteristics for those who returned study data (*n* = 87; see the “Procedure” section for further details) are presented in Table 1 (“All participants”). Psychological responses in the ED, as well as PTSD, anxiety, and depression symptoms at 1 week and 2 months, are given in Table 2 (“All participants”).

**Table 1.** Participant Characteristics for All Participants Who Returned Data (*n* = 87) and for Those Who Completed the Intrusive Memory Diary, Reporting Zero (*n* = 17) or Some (One or More; *n* = 67) Intrusive Memories (*n* = 84)

	All participants (*n* = 87)		Zero intrusive memories (*n* = 17)		Some intrusive memories (*n* = 67)		Test value (zero vs some intrusive memories)	*p*
Demographic characteristics	Mean (*SD*)	n*	Mean (*SD*)	n*	Mean (*SD*)	n*		
Age (in years)	37.86 (15.21)	87	44.06 (17.23)	17	35.33 (13.71)	67	−1.98^†^	0.047
	*n* (%)		*n* (%)		*n* (%)			
Gender (female)	41 (47.13)	87	5 (29.41)	17	35 (52.24)	67	2.83^‡^	0.092
Ethnic group (White British)	69 (79.31)	87	13 (76.47)	17	54 (80.60)	67	0.14^‡^	0.705
Marital status (married/cohabiting)	49 (56.32)	87	13 (76.47)	17	35 (52.24)	67	3.25^‡^	0.071
Employment (employed)	76 (88.37)	86	15 (88.24)	17	59 (88.06)	67	0.00^‡^	0.984
Education (higher education)	47 (55.29)	85	11 (64.71)	17	34 (51.52)	66	0.95^‡^	0.330
Pre-trauma sleep and mental health	Mean (*SD*)		Mean (*SD*)		Mean (*SD*)			
Pre-trauma sleep quality (PSQI)	4.96 (2.93)	84	5.18 (2.38)	17	4.85 (3.05)	66	−0.83^†^	0.405
	*n* (%)		*n* (%)		*n* (%)			
Sleep disorder	14 (16.28)	86	3 (17.65)	17	10 (14.93)	67	0.08^‡^	0.782
Current/past mental illness	27 (31.03)	87	6 (35.29)	17	21 (31.34)	67	0.10^‡^	0.755
Family history of mental illness	23 (26.74)	86	6 (35.29)	17	17 (25.37)	67	0.67^‡^	0.413
Prior psychological trauma	57 (66.28)	86	13 (76.47)	17	43 (64.18)	67	0.92^‡^	0.337
Details of the traumatic event								
Type of trauma								
Road traffic accident	78 (89.66)	87	15 (88.24)	17	61 (91.04)	67	0.12^‡^	0.725
Traumatic fall	6 (6.90)	87	2 (11.76)	17	3 (4.48)	67	1.29^‡^	0.257
Assault	1 (1.15)	87	0 (0.00)	17	1 (1.49)	67	0.26^‡^	0.612
Other injury	2 (2.30)	87	0 (0.00)	17	2 (2.99)	67	0.52^‡^	0.471
Brought in by ambulance	49 (56.32)	87	10 (58.82)	17	37 (55.22)	67	0.07^‡^	0.789
	Mean (*SD*)		Mean (*SD*)		Mean (*SD*)			
Time of trauma (clock time)	09:24 (2:19)	87	09:38 (2:21)	17	09:26 (2:17)	67	−0.20^†^	0.841
Pulse rate in ambulance	83.25 (17.59)	44^§^	74.67 (15.18)	9	84.79 (17.50)	34	−1.30^†^	0.194
Injury severity score	2.84 (2.96)	87	3.59 (3.36)	17	2.63 (2.78)	67	−1.20^†^	0.231
Treatment following the traumatic event	*n* (%)		*n* (%)		*n* (%)			
Admitted as inpatient	8 (9.20)	87	2 (11.76)	17	5 (7.46)	67	0.33^‡^	0.567
Medication administered in ED								
Opioids	33 (37.93)	87	6 (35.29)	17	25 (37.31)	67	0.02^‡^	0.878
Benzodiazepines	0 (0.00)	87	0 (0.00)	17	0 (0.00)	67	—	—
Other medication	58 (66.67)	87	11 (64.71)	17	45 (67.2)	67	0.04^‡^	0.848
Medication prescribed in the first week								
Opioids	18 (20.69)	87	4 (23.53)	17	13 (19.40)	67	0.14^‡^	0.705
Benzodiazepines	3 (3.45)	87	1 (5.88)	17	2 (2.99)	67	0.33^‡^	0.565
Other medication	15 (17.24)	87	4 (23.53)	17	11 (16.42)	67	0.47^‡^	0.494

*Number of participants who completed each measure.

^†^Mann–Whitney U test *z* score.

^‡^Pearson’s chi-squared test.

^§^Of 49 participants who were brought in by ambulance.

**Table 2.** Psychological Responses Following the Traumatic Event for All Participants Who Returned Data (*n* = 87) and for Those Who Completed the Intrusive Memory Diary, Reporting Zero (*n* = 17) or Some (One or More; *n* = 67) Intrusive Memories (Total *n* = 84).

	All participants (*n* = 87)		Zero intrusive memories (*n* = 17)		Some intrusive memories (*n* = 67)		Test value (zero vs. some intrusive memories)	*p*
In the Emergency Department	Mean (*SD*)	n*	Mean (*SD*)	n*	Mean (*SD*)	n*		
Perceived life threat to self	4.83 (3.42)	86	3.82 (2.88)	17	5.09 (3.48)	67	−1.38^†^	0.171
Perceived life threat to someone else	2.26 (3.44)	86	1.47 (2.40)	17	2.52 (3.67)	67	−0.89^‡^	0.374
ISRC score	5.80 (3.80)	84	3.24 (3.40)	17	6.45 (3.63)	67	−3.30^†^	0.001
PDEQ score	18.36 (7.66)	86	15.29 (4.58)	17	19.36 (8.09)	67	−1.92^‡^	0.055
PDI score	17.35 (10.46)	86	11.12 (7.75)	17	19.18 (10.46)	67	−3.02^‡^	0.003
In the first week								
Total number of intrusive memories	12.88 (18.83)	84	0.00 (0.00)	17	16.14 (19.81)	67	−6.37^‡^	<0.001
At 1 week								
Impact of Event Scale−Revised								
Intrusion	10.47 (6.52)	86	4.76 (2.86)	17	12.07 (6.38)	67	−7.00^†^	<0.001
Avoidance	8.43 (6.87)	86	4.12 (5.51)	17	9.63 (6.82)	67	−3.40^‡^	0.001
Hyperarousal	7.96 (6.52)	86	3.71 (3.00)	17	9.08 (6.82)	67	−4.86^†^	<0.001
Total	26.85 (17.68)	86	12.59 (9.00)	17	30.78 (17.62)	67	−4.25^‡^	<0.001
Hospital Anxiety and Depression Scale								
Anxiety	7.12 (4.81)	86	4.82 (3.34)	17	7.84 (4.94)	67	−2.38^†^	0.020
Depression	4.65 (4.20)	86	2.71 (3.02)	17	5.13 (4.33)	67	−2.17^†^	0.033
At 2 months								
Impact of Event Scale—Revised								
Intrusion	5.62 (5.99)	71	3.38 (3.18)	13	6.14 (6.45)	56	−1.26^‡^	0.208
Avoidance	5.65 (6.94)	71	1.77 (3.03)	13	6.61 (7.38)	56	−3.00^‡^	0.003
Hyperarousal	5.15 (5.80)	71	2.77 (2.95)	13	5.64 (6.25)	56	−1.34^‡^	0.182
Total	16.42 (17.27)	71	7.92 (6.79)	13	18.39 (18.60)	56	−1.78^‡^	0.076
Hospital Anxiety and Depression Scale								
Anxiety	5.11 (4.10)	71	3.15 (3.67)	13	5.55 (4.15)	56	−1.92^†^	0.060
Depression	3.14 (3.93)	71	2.15 (3.02)	13	3.21 (4.03)	56	−1.11^‡^	0.267
CAPS: severity scores								
Intrusion symptoms	2.40 (2.65)	80	1.33 (2.55)	15	2.60 (2.56)	63	−2.42^‡^	0.015
Avoidance symptoms	1.04 (1.44)	80	0.20 (0.56)	15	1.24 (1.53)	63	−2.71^‡^	0.007
Cognitions and mood symptoms	2.84 (3.91)	80	1.00 (1.56)	15	3.24 (4.17)	63	−2.21^‡^	0.027
Arousal and reactivity symptoms	3.28 (3.01)	80	2.60 (2.59)	15	3.44 (3.14)	63	−0.83^‡^	0.405
Total	9.55 (9.11)	80	5.13 (4.88)	15	10.53 (9.55)	63	−2.07^‡^	0.038
	*n* (%)		*n* (%)		*n* (%)			
CAPS: PTSD diagnostic criteria	10.00 (12.50)	80	0.00 (0.00)	15	10.00 (15.87)	63	2.73^§^	0.098

*Number of participants who completed each measure.

^†^ *t*-test.

^‡^Mann–Whitney U test *z* score.

^§^Pearson’s chi-squared test.

### Procedure

Ethical approval for the study was obtained from East Midlands—Derby National Health Service Research Ethics Committee (reference number: 16/EM/0326). The study was registered in a public trials registry before commencing recruitment (ClinicalTrials.gov Identifier: NCT03012685). After providing written consent to take part in the study, participants were given a study booklet, containing all measures to be completed while in the ED (demographics, physical and mental health, perceived life threat, Peritraumatic Dissociative Experiences Questionnaire-Self Report [PDEQ] [39], Peritraumatic Distress Inventory [PDI] [40], Immediate Stress Reaction Checklist [ISRC] [41], Pittsburgh Sleep Quality Index [PSQI] [42]); during the first week after the trauma (daily sleep and intrusive memory diary); and at the end of 1 week (Impact of Event Scale—Revised [IES-R] [43], Hospital Anxiety and Depression Scale [HADS] [44]). Eighty-seven participants (88%) returned the study booklet. Details of the traumatic event and treatment received following the traumatic event (including inpatient admission and medication administered) were collected from medical records following patient discharge. The severity of the physical injury, indicated by the Injury Severity Score (ISS) (range 0–75), was rated from medical records using the Abbreviated Injury Scale [45]. Participants were also given the option of wearing an actigraph (a watch-like device assessing activity levels) for the first week after the traumatic event (*n* = 90 took actigraphy; *n* = 80, 81%, completed actigraphy). Participants were sent twice-daily automated text messages (morning and evening) reminding them to fill in the diary and were telephoned by a study investigator at the beginning and end of the first week to reinforce understanding of the study procedure. A study investigator clarified any unclear responses in the study booklet with the participant via telephone where possible. Two months after the traumatic event, participants were contacted by telephone to complete the Clinician-Administered PTSD Scale (CAPS; *n* = 80, 81%, completed) and asked to complete additional self-report questionnaires online or by post (IES-R and HADS; *n* = 71, 72% completed). After receiving the final questionnaires, participants were sent debriefing information about the study by email or post and reimbursed up to £30 (GBP) for their time. A participant flow diagram is shown in Supplementary Figure S1). Although sleep was also assessed in the first week following the traumatic event, only sleep on the first night is included in this analysis.

### Measures

#### Participant characteristics

A self-report questionnaire assessed demographic characteristics, current sleep disorder, current or past mental health problems, family history of mental health problems, and previous experience of psychological trauma.

#### Perceived life threat

Two items were used to assess perceived life threat during the trauma: “to what extent did you feel your life was in danger?” and “to what extent did you feel that someone else’s life was in danger?” (based on Ref. [46])—each rated from 0 (not at all) to 10 (extremely).

#### Peritraumatic Dissociative Experiences Questionnaire-Self Report

Dissociative symptoms during the trauma were assessed using the PDEQ, a 10-item scale, with a summed global score of 10–50 [39].

#### Peritraumatic Distress Inventory

Emotional responses during the trauma were assessed using the PDI, a 13-item scale, which gives a summed total score of 0–52 [40].

#### Immediate Stress Reaction Checklist

Acute stress responses immediately after the trauma were assessed using the re-experiencing, avoidance and hyperarousal symptom items of the ISRC, with a summed total score ranging 0–18 [41].

#### Pittsburgh Sleep Quality Index

Self-reported sleep quality for the month before the trauma, was assessed with this nine-item scale, which gives a global score of 0–21, with a score of 5 or more being indicative of poor sleep [42].

#### Sleep diary

The primary measure of sleep duration was a pen-and-paper self-report sleep diary [47]. Participants were asked to record what time they went to bed (sleep time), how long it took them to fall asleep (sleep latency), what time they woke up (wake time), and how many times they woke during the night and for how long (wake after sleep onset). If any of these items were not completed, sleep duration could not be calculated.

#### Actigraphy

Participants who opted to wear an actigraph (MotionWare 8; CamNtech Ltd, Cambridge, UK) were instructed to wear it on their non-dominant wrist, though the dominant wrist was used if the non-dominant arm/wrist was injured. Actigraphy data were sampled in 1 min epochs and analyzed with MotionWare software (version 1.2.14, CamNtech Ltd). Activity levels were annotated using the sleep diary data: “bedtime” and “getup time” were manually entered and “sleep start” and “sleep end” calculated automatically using high sensitivity.

#### Intrusive memory diary

Intrusive memories were recorded in a daily pen-and-paper diary (adapted from Refs [48–50]) for the first week after the trauma (day 1 to day 8). Participants recorded the occurrence of an intrusive memory by ticking a box for the day and time period (getup time to midday, midday to 05:00 pm, 05:00 pm to bedtime, during the night) on which it occurred or marked “zero” if none were experienced. Intrusive memories were defined in the study booklet as “image-based memories of the incident that pop into your mind without warning. They often take the form of visual pictures in your mind’s eye e.g. like a snapshot image or film clip. They can vary from being vivid and emotional to being very short, fleeting and broken up. They can also include other senses e.g. sounds and smells such as the smell of smoke. They may or may not be triggered by something you are aware of e.g. telling someone about the incident, being back at the scene. They may include fully reliving the incident, and acting or feeling as if it was happening again, which tends to be less common. You may also have nightmares of the incident, these are intrusive memories as well.” Participants were instructed not to record memories that were deliberately recalled or general thoughts about the incident without a mental image. For participants who completed each day of the intrusive memory diary (*n* = 84), the sum number of intrusive memories was calculated for each day and a total number for the week from the day after the trauma (i.e. day 2 to day 8). The day of the traumatic event (day 1) was not included as this preceded sleep on the first night.

#### Impact of Event Scale—Revised

The IES-R was used to assess the self-reported impact of the trauma [43]. This 22-item scale assesses symptoms over the previous 7 days. Items were summed for intrusion, avoidance, and hyperarousal subscales and to give a total score ranging from 0 to 88.

#### Hospital Anxiety and Depression Scale

The HADS was used to assess anxiety and depression symptoms [44]. It is a 14-item scale (seven items each for anxiety and depression subscales). Each item is scored from 0 to 3, and a summed score ranging from 0 to 21 is calculated for each subscale.

#### Clinician-Administered PTSD Scale for DSM-5 (CAPS-5 [51])

PTSD symptom severity and diagnosis were assessed about 2 months (mean 67.5 days, *SD* 20.01, range 53–196) following the traumatic event using the CAPS-5 [51]. The CAPS-5 is a 30-item structured clinical interview, with a symptom severity score ranging from 0 to 80. Interviews were conducted via telephone, which has been shown to be as reliable as face-to-face interviews [52] and digitally recorded. A randomly selected 10% of all CAPS interviews were rated by an independent assessor who was blind to the original scoring. Interrater agreement for PTSD diagnosis was 87.5%, and the intraclass correlation coefficient for the total severity score was 0.82, indicating good reliability [53].

### Data analysis

#### Sleep duration calculation

Sleep duration was calculated from self-reported sleep timings from the sleep diary: [(wake time − sleep time) − (sleep latency + wake after sleep onset)]. The primary outcome was sleep duration on the first night after the trauma (SD_N1). In addition, to investigate sleep duration relative to participants’ habitual sleep duration, the change in sleep duration from before the trauma to the first night after the trauma was calculated using question 4 of the PSQI: SD_change = [(sleep duration on first night from sleep diary) − (sleep duration before trauma from PSQI)].

A sleep diary, as opposed to actigraphy, was chosen as the primary measure of sleep duration to limit the confound of participants having restricted movement due to potential physical injuries sustained during the traumatic event. As seen with Insomnia Disorder [54, 55], this lack of movement could over-inflate the duration of sleep. Actigraphy, however, was used as an optional, additional measure of sleep duration to enable comparison of sleep diary and actigraphy in this population. A moderate positive correlation was found between sleep duration on the first night post-trauma from the sleep diary and from actigraphy (Pearson *r* = 0.462, *p* < 0.001, *n* = 71), which is consistent with correlations seen within a cohort including those with Insomnia Disorder [55].

### Power analysis

A medium effect size of *r* = 0.3 [37] was estimated for the correlation coefficient between sleep duration and the total number of intrusive memories, based on the work of Porcheret et al. [27], who found a medium effect size of Cohen’s *d* = 0.47 on the number of intrusive memories after experimental trauma. Based on this, the study required 85 participants for 80% power at the 5% significance level, two-tailed. The sample size was increased to 100 participants to allow for 15% attrition.

### Management of missing data

Missing questionnaire items were pro-rated from other items in the same subscale/questionnaire where possible (i.e. mean of other items in the same subscale/questionnaire; 4 data points). Imprecisely reported sleep data (e.g. “a few minutes”) were recorded as an estimated value where appropriate (e.g. 5 min) (22 data points) or recorded as missing if an estimated value was not appropriate (e.g. “ages”) (34 data points). When imprecise intrusive memory data indicating a high number were reported (e.g. “all the time”), the highest value of intrusive memories reported by any participant for one time period was recorded (2 data points). For a missing time of trauma, time admitted to the ED was used (six participants).

### Statistical Analysis

Statistical analyses were performed on the available data for each variable (sample sizes are given in Tables 1 and 2). Continuous data were assessed for normality by examining histograms and statistics of skewness and kurtosis [56]. Parametric or nonparametric tests were used as appropriate. Between-group comparisons (*t*-test or Mann–Whitney U-test for continuous data; chi-squared test for categorical data) and bivariate correlations (Pearson’s or Spearman’s) were performed using SPSS version 25.

To investigate the relationship between sleep duration on the first night following the traumatic event and the total number of intrusive memories reported in the subsequent week (day 2 to day 8), we used a mixture model [57] (in this case a zero-inflated Poisson distributed model), to deal with the greater than expected (overinflation of) zero counts in the intrusive memory data. A mixture model derives the properties of the overall population from subpopulations within the data. This approach allowed non-zero counts (one or more intrusive memories) to be modeled with a Poisson distribution, and zero counts (no intrusive memories) with a Binomial distribution within one model using the following equation:

$$\begin{array}{l} \mathrm{Pr}\left(IM=j\right)=\left\{ \begin{array}{l} p+\left(1-p\right)\exp\left(-u\right)\;if\;j=0\\ (1-p)\frac{u^{IM}exp(u)}{IM!}\;if\;j=0 \end{array}\right.\frac{n!}{r!\left(n-r\right)!} \end{array}$$

where the probability of observing *j* intrusive memories (Pr(IM = *j*)) is split depending on whether *j* = 0 or *j* > 0; different distributions are used to describe the probability of zeros (*j* = 0; a combination of a Binomial distribution and the zeroth term of a Poisson distribution) and the probability of not zeros (*j* > 0; a Poisson distribution), where *p* is the probability of zero intrusive memories (which can occur because of a true count of zero or through other processes) and *u* is the expected (average) number of intrusive memories. The same model was used to investigate the relationship between the change in sleep duration from before the trauma to the first night after the trauma and the total number of intrusive memories in the subsequent week.

To investigate the temporal patterns of intrusive memories over the week, we used the mixture model framework to investigate how the number of intrusive memories (IM) on a given day (*t*) was influenced by the (nonlinear relationship of the) number of intrusive memories on the previous day (*t*−1) and sleep (*S*) on the previous day. The full statistical model for the time series analysis is

$$\begin{array}{l} IM_{t}=IM_{t-1}+{\left(IM_{t-1}\right)}^{2}+S_{t-1} \end{array}$$

Mixture models and time series analyses were performed in R [58].

To explore the relationship between sleep duration on the first night and the total number of intrusive memories in the first week with PTSD at 2 months, we used between-groups analyses to compare participants who did and did not meet CAPS PTSD criteria at 2 months on intrusive memories in the first week and sleep duration on the first night.

In line with the study of Tabachnick and Fidell [56], and as in previous studies assessing the number of intrusive memories following real or analogue trauma exposure (e.g. the studies of Iyadurai et al. [38] and Lau-Zhu et al. [59], respectively), a data point that was a statistical outlier (defined as those with standardized *z* scores >3.29), but considered to be a real value from the intended population, was retained in the analysis alongside taking a statistical approach to reduce the impact of such cases. In the study of Iyadurai et al. [38], this was achieved by log transformation of the data and using a *t*-test. In our data, four statistical outliers were identified for the number of intrusive memories and were considered to be from the intended population for the following reasons: values were consistent with those in a previous study of patients recruited from a hospital ED (Ref. [38], control group); the current study used an unselected population of ED patients exposed to trauma (rather than only those with or without a clinical diagnosis), hence a large variation including some extreme values was to be expected; values were not excessively higher than other high values in the sample; and the four cases were not outliers on other variables, suggesting the participants were not unrepresentative of the population. Our analysis used a mixture model that does not assume a normal distribution and was in fact fitted due to the heterogeneity in the data.

## Results

### Sleep duration

On the first night after the traumatic event, the mean sleep duration from the sleep diary was 6.98 h (*SD* 2.33, range 1–12.5, *n* = 77). The mean sleep duration prior to the traumatic event was 7.00 h (*SD* 1.09, range 3.5–10, *n =* 85) and the mean change in sleep duration from pre- to post-trauma was −0.42 h (*SD* 2.29, range 0–2.93, *n* = 77). There was no significant difference in sleep duration from pre- to post-trauma (paired *t*-test: *t* = 0.263, *p* = 0.793). Of note, no participants reported complete sleep deprivation on the first night from either the sleep diary or actigraphy.

### Intrusive memories

**Figure 1.**
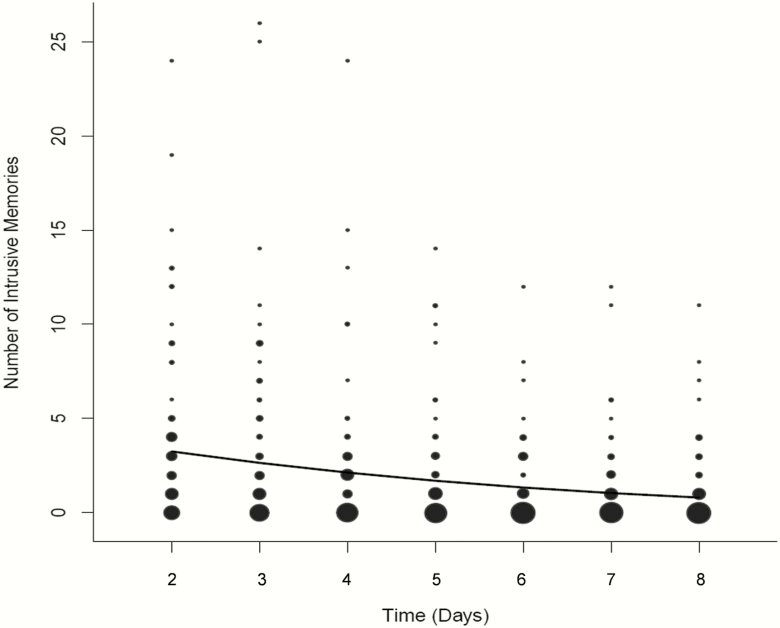
Frequency scattergraph showing the time course of the number of intrusive memories per day for the first week after the day of the traumatic event (day 2 to day 8). The size of the circles represents the number of participants who reported the indicated number of intrusive memories on that particular day. The solid line shows the fit of a mixture model to summarize the number of intrusive memories through the 7-day period.

A mean of 12.88 intrusive memories (*SD* 18.83, range 0–104, *n* = 84) was reported over the week (from the day after the trauma, which is day 2, to day 8). The time series analysis showed a significant decline in the number of intrusive memories over the course of the week: with the number of intrusive memories on one day predicting fewer the next (from day 2 to day 8; Figure 1). For the non-zero counts the best fit time series model was IM_*t*_ = exp (0.045 (*SE* = 0.093) – 0.257 (0.019) IM_*t*−1_ – 0.006 (0.001) (IM_*t*−1_)^2^). For the zero counts the best-fit time series model was IM_*t*_ = logit (1.374 (*SE* = 0.220) – 2.366 (0.461) IM_*t*−1_ + 0.091 (0.018) (IM_*t*−1_)^2^). Sleep on the previous night did not have a significant effect for either the non-zero counts (estimate −0.003 (*SE* = 0.018), *z* = 0.160, *p* = 0.873) or the zero counts of intrusive memories on a given day (estimate −0.069 (*SE* = 0.100), *z* = 0.692, *p* = 0.489).

### Relationship between sleep duration and intrusive memories

**Figure 2.**
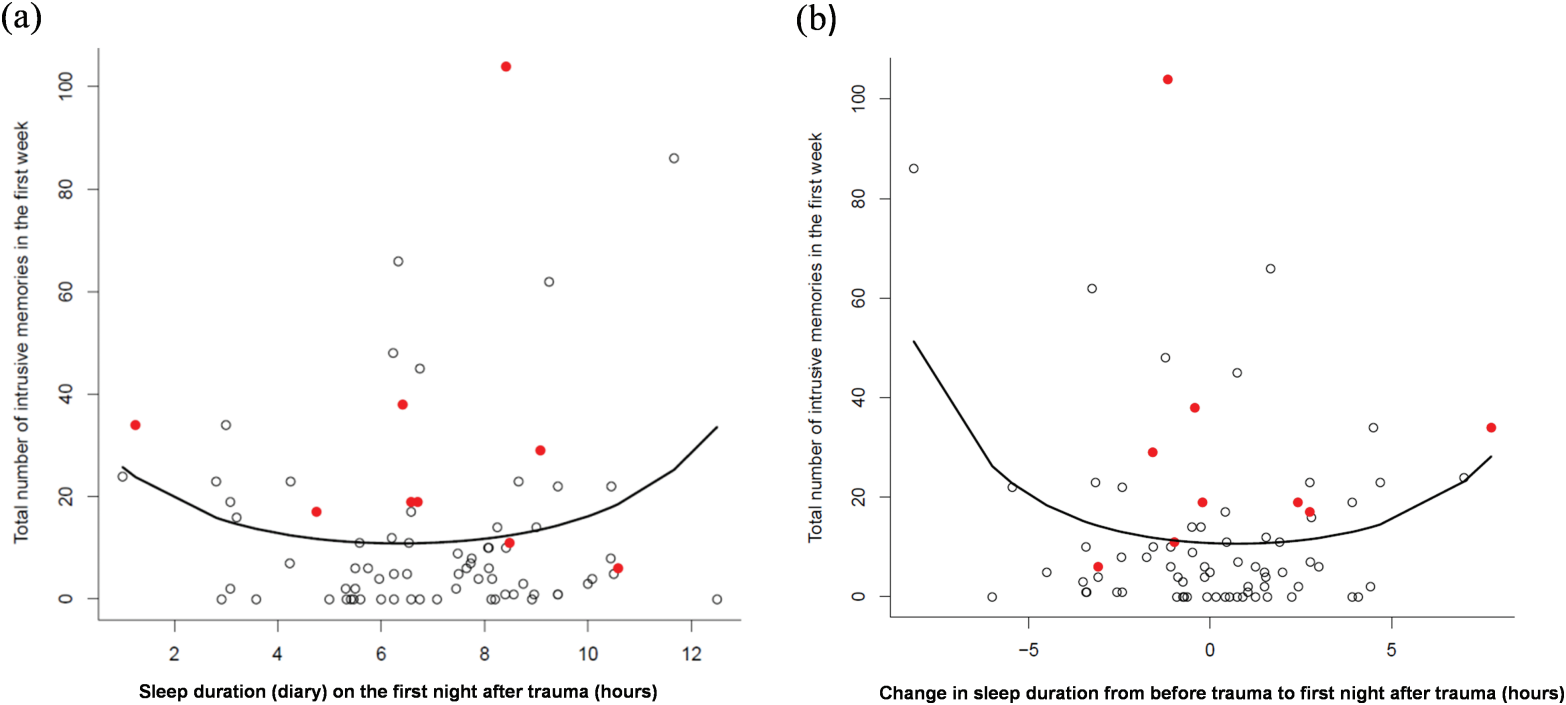
Scattergraphs showing the relationship between (A) sleep duration on the first night after the traumatic event (sleep diary) and the total number of intrusive memories in the subsequent week (day 2 to day 8), and (B) change in sleep duration from before the trauma to the first night after the trauma and the total number of intrusive memories in the subsequent week (day 2 to day 8). The lines show significant positive quadratic relationships, indicating that (A) a “shorter” and “longer” sleep duration was associated with more intrusive memories than an intermediate sleep duration, and (B) a “decrease” and “increase” in sleep duration was associated with more intrusive memories than no change in sleep duration from before the traumatic event. Participants who met CAPS criteria for PTSD at 2 months are marked in red.

The mixture model revealed a significant positive quadratic (U-shaped) relationship between sleep duration on the first night (sleep diary; SD_N1) and the total number of intrusive memories in the subsequent week for non-zero counts, that is one or more intrusive memories (total IMs = exp (3.384 (*SE* = 0.149) – 0.382 (0.049) SD_N1 + 0.030 (0.004) (SD_N1)^2^), *n* = 76; Figure 2, A), and a nonsignificant (intercept-only) relationship for zero counts, that is no intrusive memories (SD_N1 estimate 0.202 (*SE* = 0.557), *z* = 0.362, *p* = 0.717). This intercept-only relationship for zero counts highlights that the proportion of zeros is the best descriptor of the (over-inflated) zero counts (and it is not necessary to include any additional covariates). Derived from the mixture model, in the non-zero group, the shortest (1 h), 25% quantile (4 h), median (6.9 h), 75% quantile (9.7 h), and longest (12.5 h) sleep durations were associated with 20.74, 10.53, 8.80, 12.02, and 26.78 expected intrusive memories, respectively. The mixture model was a sufficient fit without the need for additional covariates.

Sleep duration on the first night was also assessed relative to sleep duration prior to the traumatic event (SD_change). From the mixture model, a significant positive quadratic relationship was found for the relationship between the change in sleep duration pre-trauma to sleep duration on the first night after the trauma and the total number of intrusive memories in the subsequent week for non-zero counts (total IMs = exp (2.614 (*SE* = 0.038) – 0.029 (0.009) SD_change + 0.020 (0.002) (SD_change)^2^), *n* = 76; Figure 2, B) and a nonsignificant effect (intercept-only relationship) for zero counts (estimate 0.086 [*SE* = 0.129], *z* = 0.665, *p* = 0.506). Thus, an increase or decrease in sleep duration from pre-trauma to the first night post-trauma was associated with more intrusive memories compared to no change in sleep duration. From this mixture model, for the non-zero group, the predicted number of intrusive memories for the lowest extreme, 25% quantile, median, 75% quantile, and highest extreme in the differences in sleep were 65.75, 14.69, 13.67, 13.72, and 36.25, respectively.

### Relationship of intrusive memories and sleep duration with CAPS assessment of PTSD at 2 months

At 2 months, 10 participants were found to have a CAPS score indicating they met the criteria for a diagnosis of PTSD, shown in red in Figure 2. All participants who met CAPS PTSD criteria (PTSD+) had some intrusive memories in the first week (i.e. none had zero) and reported on average more intrusive memories (day 2 to day 8) than participants who did not meet CAPS PTSD criteria (PTSD−; PTSD + mean = 28.20, *SD* = 28.86, range = 5–104, *n* = 10; PSTD − mean = 9.96, *SD* = 13.88, range = 0–66, *n* = 68; Mann–Whitney U *z* = −3.09, *p* = 0.002, *n* = 68). No difference was seen in mean sleep duration (sleep diary) on the first night after the traumatic event or the change in sleep duration from pre- to post-trauma between participants who did and did not meet CAPS PTSD criteria (first night sleep duration: PTSD + mean = 6.92 h, *SD* = 2.74, *n* = 9; PTSD − mean = 7.03, *SD* = 2.25, *n* = 63; Mann–Whitney U *z* = −0.25, *p* = 0.805, *n* = 72; change in sleep duration: PTSD + mean = −0.61 h, *SD* = 3.25, *n* = 9; PTSD − mean = −0.4 h, *SD* = 2.57, *n* = 63; Mann−Whitney U *z* = −0.034, *p* = 0.973).

### Post hoc analyses: participants with zero versus some intrusive memories

As participants with zero intrusive memories and those with some (one or more) intrusive memories were modeled separately in the mixture model, post hoc analyses were conducted to examine differences in participant characteristics and psychological responses to the trauma between these two groups (see Table 1 and Table 2). Participants with some intrusive memories (*n* = 67), compared to those with zero intrusive memories (*n* = 17), were younger (see Table 1). They also had a greater immediate stress response (ISRC score) and emotional response (PDI score) in the ED; greater posttraumatic stress symptoms (IES-R scores) and anxiety and depression symptoms (HADS scores) at 1 week; and greater PTSD symptom severity (CAPS severity scores for intrusion, avoidance, and cognitions and mood symptoms and total CAPS severity score) and avoidance symptoms (IES-R avoidance subscale score) at 2 months (see Table 2). No difference was found for the other IES-R subscales (intrusion and hyperarousal) or total score, or for the arousal and reactivity symptom severity score on the CAPS at 2 months.

## Discussion

This study provides prospective, empirical assessment of sleep duration (on the first night) and intrusive memories (in the subsequent week) immediately after a psychologically traumatic event (one that met DSM-5 criteria, e.g. a traumatic motor vehicle accident, see Table 1) and later PTSD symptoms (at 2 months).

### Sleep duration on the first night and intrusive memories in the subsequent week

A positive quadratic (U-shaped) relationship was found between sleep duration on the first night after the traumatic event and the number of intrusive memories of that event over the subsequent week: sleeping “too little” or “too much” was associated with having more intrusive memories. Thus, a long (at the extreme 12.5 h) or short (at the extreme 1 h) sleep duration, compared to an intermediate sleep duration (6.9 h), was associated with having 26.78 and 20.74, compared to 8.80, intrusive memories, respectively. A similar pattern was seen when examining the change in sleep duration from pre- to post-trauma, with either an increase or a decrease in sleep duration being associated with more intrusive memories compared to no or little change. This suggests that the *change* in sleep duration compared to usual may be important as well as the overall duration of sleep in influencing the number of intrusive memories.

Our results extend findings showing that sleep disturbances *in the weeks* following a real-life traumatic event are associated with early intrusive memories [36] by showing that the disruption of sleep *from the very first night* following a traumatic event may be associated with increased early posttraumatic symptoms. Direct comparison with experimental studies is limited, as we observed natural variation in sleep duration as opposed to distinct forced wake or sleep periods [26–32]. Since total sleep deprivation was not seen in this clinical study, less extreme sleep schedules (reflecting natural variation in sleep duration) should be considered in future experimental studies. In comparison with the wider literature, the relationship between sleep duration and other health outcomes also often shows a U-shaped relationship; both long and short sleep durations have been associated with worse physical and mental health outcomes [60–62]. In contrast to sleep disturbance in the weeks and months following a real-world traumatic event [33–35], no association was found in this study between sleep duration on the first night following a traumatic event and later PTSD. The reason for this could be that sleep on the first night is not associated with the development of PTSD, or alternatively that its effect is too small to be detected in this population, possibly as a result of limited numbers of participants with extreme sleep durations. Further exploration of the extremes of sleep duration on the first night following a traumatic event could help clarify this issue.

### Intrusive memories in the first week and CAPS assessment of PTSD at 2 months

The number of intrusive memories in the first week after trauma was approximately three times higher in participants meeting the CAPS criteria for PTSD [51] at 2 months compared with those who did not. All participants meeting CAPS criteria for PTSD at 2 months reported a total of five or more intrusive memories in the first week, that is, none reported zero. These findings offer a first indication of the quantification of the relationship between the *number* of intrusive memories in the first week after trauma and the presence of later PTSD in this population. Examined another way, the absence of intrusive memories in the first week (seen in 20% of participants with intrusive memory data) was only observed in those who did not go on to later meet CAPS criteria for PTSD.

Furthermore, post hoc analyses found that participants with zero intrusive memories had lower CAPS severity scores for all PTSD symptom clusters apart from arousal symptoms at 2 months (although we note there was not a corresponding difference in scores on the IES-R intrusion subscale at 2 months, possibly reflecting the different time scales of the IES-R [past week] and CAPS [past month]). These findings must of course be treated cautiously as this study was not powered to look at predictive factors for the development of PTSD, and with only 10 participants meeting CAPS criteria for PTSD at 2 months this study has a limited sample size. Nevertheless, the strength of these observations merits further investigation and is perhaps more clinically striking than the findings on sleep duration.

A relationship between the severity of early intrusive memories (mean of 7 days post-trauma), assessed using a retrospective clinical interview, and later PTSD has been reported in previous research with injured trauma survivors [63]. Our results extend previous findings by showing that the number of intrusive memories in the first week specifically, assessed prospectively from the day after the trauma, is associated with subsequent PTSD symptomatology.

Another consideration regarding intrusive memories in the first week and the later development of PTSD symptoms are the other factors known to predict PTSD, in particular immediate psychological reactions to the trauma such as perceived life threat, peritraumatic distress, and peritraumatic dissociation. In this study, perceived life threat was not found to differ between participants who reported some intrusive memories and those who reported zero intrusive memories, while both ISRC and PDI scores were higher in those who reported some intrusive memories. Furthermore, participants who experienced some intrusive memories, compared to none, were found to be younger on average; but other demographics, pre-trauma sleep and mental health, type and severity (as assessed by ISS) of the trauma, and medication were not found to differ between the groups. The influence of such factors should be considered in future studies.

### Limitations

We recognize several limitations to this study. First, although retention was good (88%), it was not possible to examine if the participants who did not return their study booklet differed from the rest of the sample, as baseline data were only recorded in the study booklet. Future studies should collect baseline data before participants leave the hospital. Second, other factors could have affected sleep (e.g. medication, admission to hospital, injury severity), and this could be explored in future studies. Third, although the assessment of sleep before the traumatic event was only possible retrospectively, the use of one item from the PSQI as a comparison to the prospective sleep diary is a limitation in this study. Fourth, even though all types of trauma were eligible for inclusion in the study, road traffic accidents were the most common, limiting generalization to other trauma types. Recruitment was limited to core working hours of the research nurse team, so traumas occurring in the evening and weekends were missed. However, measures of trauma exposure (e.g. perceived life threat, peritraumatic distress) were comparable to other studies of injured patients recruited from a hospital ED [38, 46]. Fifth, as the study was powered on the assumption of a linear relationship between sleep duration and intrusive memory frequency, we lacked sufficient numbers to examine PTSD rates in participants with extreme (long or short) compared to intermediate sleep durations. Larger studies might seek to examine this, as well as the relative impact of sleep and intrusive memories on PTSD, controlling for other known predictive factors (e.g. peritraumatic distress, peritraumatic dissociation), including age (which was found to differ between those with no vs some intrusive memories). Furthermore, the final sample size for the primary analysis was fewer than required (76 instead of the 85 estimated by the power calculation), hence the obtained power for the primary analysis was approximately 74% compared to the originally anticipated 80%. Finally, sleep characteristics other than sleep duration, for example, nighttime awakenings or REM sleep periods specifically, may be important in the development of intrusive memories, and this needs to be explored further.

### Implications

A notable outcome of this study is that participants who met CAPS criteria for PTSD 2 months post-trauma had *three times more intrusive memories* in the first week post-trauma compared to participants who did not (*M* = 28.20 vs 9.96), and that all these participants reported at least five intrusive memories in this first week. The clinical implication of this is that monitoring intrusive memories in the first week post-trauma, with even a simple diary, may help identify people more likely to be in need of either early intervention or follow-up support. The implication for future research is that intrusive memories might be one potential target for developing early interventions after trauma. One approach to targeting intrusive memories is a brief behavioral procedure, involving a memory reminder cue followed by a visual interference task (playing the computer game Tetris), delivered within the first few hours post-trauma [49]: recent proof-of-concept trials show a reduction in the number of intrusive memories in the first week [18, 19]. Conversely, the absence of this core symptom as early as the first week after a traumatic event (i.e. *zero intrusive memories* as seen in 20% of the current sample) may identify those at low risk of later possible PTSD, who do not require early intervention. In this study, no participants with zero intrusive memories in the first week post-trauma went on to meet the CAPS criteria for PTSD at 2 months.

Regarding sleep duration immediately after the traumatic event, a very marked *change* in usual sleep duration on the first night post-trauma, both longer and shorter, was associated with developing more intrusive memories. Determination of the precise amount of sleep that is “too little” or “too much” for an individual following a traumatic event will require more extensive research. However, there remains possible clinical relevance for assessing patients in the immediate aftermath of trauma, for example, in acute hospital wards, where patients are already monitored overnight: asking them about sleep duration on the first night after the trauma, and if this has markedly changed compared to usual, may identify those more vulnerable to subsequent symptoms. Simple methods of monitoring sleep, and monitoring intrusive memories, such as using a self-report diary, might even hold potential for identifying those at risk of later psychopathology in the immediate aftermath of widespread global trauma such as terrorist attacks and natural disasters.

## Supplementary Material

zsaa033_suppl_Supplemental_material

## Deposit of Material in Data Repository

Data will be made publically available via Open Science Framework at https://osf.io/fsn2f/ (with the exception of potentially identifying patient information).

## Notes

This work was conducted at the Nuffield Department of Clinical Neuroscience and Department of Psychiatry, University of Oxford and the Emergency Department, Oxford University Hospitals NHS Foundation Trust, Oxford.
