# Supplementary material for: Sleep and intrusive memories immediately after a traumatic event in emergency department patients
Source: Sleep. 2020 Mar 5;43(8):zsaa033. doi: 10.1093/sleep/zsaa033 (PMC7420515; doi:10.1093/sleep/zsaa033)
Supplement: zsaa033_suppl_Supplemental_material [file zsaa033_suppl_supplemental_material.docx]

**Supplemental material**

Approached about the study

(*n* = 162)

Provided informed consent

(*n* = 100)

Eligible for inclusion

(*n* = 99)

Given study booklet

(*n* = 99)

Enrollment

Screened for eligibility

(*n* = 976)

Excluded: did not meet eligibility criteria

(*n* = 814)

Declined to participate

(*n* = 62)

Excluded: found to no longer meet eligibility criteria

(*n* = 1)

In the emergency department

Given actigraphy (optional)

(*n* = 90)

Returned study booklet

(*n* = 87)

[Did not return booklet (*n* = 10), dropped out (*n* = 2)]

Completed actigraphy

(*n* = 80)

[Did not return actigraphy (*n* = 7), did not wear actigraphy (*n* = 3)]

At one week after the trauma

Sample with complete data for sleep duration on night 1 (sleep diary) and intrusive memories

(*n* = 76)

At two months after the trauma

Completed CAPS

(*n* = 80)

Completed self-report questionnaires (*n* = 71)

**Figure S1.** Participant flow diagram. CAPS = Clinician-Administered PTSD Scale.
